# A practical synthesis of the ^13^C/^15^N-labelled tripeptide *N*-formyl-Met-Leu-Phe, useful as a reference in solid-state NMR spectroscopy

**DOI:** 10.3762/bjoc.4.35

**Published:** 2008-10-13

**Authors:** Sven T Breitung, Jakob J Lopez, Gerd Dürner, Clemens Glaubitz, Michael W Göbel, Marcel Suhartono

**Affiliations:** 1 Institute of Organic Chemistry and Chemical Biology, Johann Wolfgang Goethe University Frankfurt, Max-von-Laue-Str. 7, D-60438 Frankfurt am Main, Germany; 2 Institute of Biophysical Chemistry, Johann Wolfgang Goethe University Frankfurt, Max-von-Laue-Str. 9, D-60438 Frankfurt am Main, Germany

**Keywords:** Fmoc solid phase peptide synthesis, formylation, f-MLF, magic-angle spinning, Wang resin

## Abstract

A mild synthetic method for *N*-formyl-Met-Leu-Phe-OH (**1**) is described. After Fmoc solid phase peptide synthesis, on-bead formylation and HPLC purification, more than 30 mg of the fully ^13^C/^15^N-labelled tripeptide **1** could be isolated in a typical batch. This peptide can be easily crystallised and is therefore well suited as a standard sample for setting up solid-state NMR experiments.

## Introduction

There appears to be a general lack of widely available and standardised samples for setting up new solid-state NMR experiments. Such a standard sample should show a small ^13^C and ^15^N linewidth and a short ^1^H *T*_1_ relaxation time. It should contain a number of molecular groups with different chemical shifts for setting up correlation spectra. The ^13^C/^15^N-labelled tripeptide *N*-formyl-Met-Leu-Phe-OH (f-MLF-OH) (**1**) has been shown in a number of solid-state NMR studies to fulfil these criteria. It has been used in great detail to examine spin dynamics in peptide and for distance measurements [1–9]. MLF is a chemotactic peptide which plays an important role in antibody research [10].

Although this tripeptide was briefly commercially available in the past, only one synthesis of f-MLF-OH (**1**) by solid phase methods has been published so far [11]. Rigorous reaction conditions (EtOH, reflux, 24–65 h) were required to couple the first Boc-protected amino acid to the solid support (chloromethyl resin) and long reaction times (18 h) were necessary to attach further building blocks to the growing peptide chain. The formylation of the *N*-terminus with formic acid/acetic anhydride was carried out after cleavage from the resin with liquid hydrogen fluoride [11–12]. No experimental procedures were given in all subsequent publications. In this work, we present for the first time in detail an improved practical synthesis for fully ^13^C/^15^N-labelled f-MLF-OH (**1**) based on the Fmoc-strategy.

## Results and Discussion

### Peptide Synthesis

**Scheme 1:**
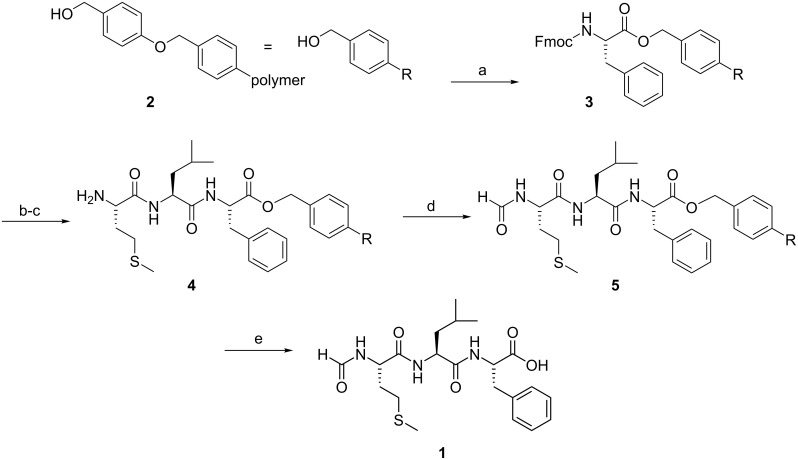
Synthesis of f-MLF-OH (**1**). a) Fmoc-Phe-OH, MSNT, MeIm, over night. b) 1. piperidine, 30 min; 2. Fmoc-Leu-OH, DIC, HOBt*H_2_O, 3 h. c) 1. piperidine, 30 min; 2. Fmoc-Met-OH, DIC, HOBt*H_2_O, 3 h; 3. piperidine, 30 min. d) HCOOEt, over night. e) TFA, EDT, H_2_O, TIS (94 : 2.5 : 2.5 : 1), 4.5 h. All reactions were carried out at room temperature. Abbreviations: MSNT = 1-(mesitylene-2-sulphonyl)-3-nitro-1,2,4-triazole, MeIm = *N*-methylimidazole, DIC = diisopropylcarbodiimide, HOBt = 1-hydroxybenzotriazole, TFA = trifluoroacetic acid, EDT = ethanedithiole, TIS = triisopropylsilane.

The synthesis of the MLF tripeptide started with the immobilisation of ^13^C/^15^N-labelled Fmoc-Phe-OH to the solid support (Wang resin **2**). This esterification step, leading to **3** quantitatively, was performed by activating the COOH group with MSNT under mild reaction conditions (Scheme 1) [13]. Full conversion of the resin bound hydroxy groups could be demonstrated by a colourimetric test with Dabcyl-COOH [14]. After removal of Fmoc with piperidine, the ^13^C/^15^N-labelled monomers Fmoc-Leu-OH and Fmoc-Met-OH were successively coupled to the solid support with DIC/HOBt [15]. Quantitative conversion in each reaction was monitored by the Kaiser test [16–17]. The resulting tripeptide **4** was formylated under mild conditions with ^13^C-labelled ethyl formate to obtain **5** [18]. The main advantage of using ethyl formate is the possibility of re-isolation of the expensive ^13^C-labelled reagent for further formylation reactions, due to the absence of activating agents. To the best of our knowledge, formylation reactions of *N*-termini with ethyl formate have never been reported before in solid phase peptide synthesis. Other methods of formylation were not tested, since they require elevated reaction temperatures or acidic conditions [19–20], which might cause some premature removal of the peptide from the resin. The final step was the cleavage of f-MLF-OH (**1**) from the solid support with TFA [21–22]. To prevent by-product formation from electrophilic intermediates present in the cleavage process, EDT and TIS were added as scavengers [23–24].

## Conclusion

Compared to the solution phase synthesis of peptides, the solid phase synthesis offers a simplified purification of the intermediates. With the improved synthetic protocol based on Fmoc building blocks, where all reaction steps were carried out at room temperature, we were able to obtain the per-^13^C/^15^N-labelled formylated Met-Leu-Phe tripeptide as carboxylic acid in acceptable yields [32.8 mg (23%) after HPLC purification]. Due to the selected reagents, i.e. MSNT for the esterification, DIC/HOBt for the peptide coupling and ethyl formate for the formylation, shorter reaction time and quantitative conversion could be accomplished when compared to the previous protocol from 1976 [11], where yields are not given. In contrast to the Boc-strategy, the Fmoc-method for solid phase peptide synthesis has the advantage of orthogonal conditions for the removal of *N*-protective groups and the cleavage from the Wang resin [25]. Unintentional release of the growing peptide chain thus could be excluded. In addition, the use of liquid hydrogen fluoride is no longer required.

## Experimental

**General:** All reagents were obtained from commercial suppliers and were used without further purification. HPLC gradient: 0–5 min (0.1% TFA/MeCN 99:1), 5–20 min (0.1% TFA/MeCN 99:1 to 30:70), 20–25 min (0.1% TFA/MeCN 30:70), 25–30 min (0.1% TFA/MeCN 30:70 to 99:1), 30–40 min (0.1% TFA/MeCN 99:1). ESI-MS: *Fisons* VG Plattform II. NMR: *Bruker* AM 300 (^1^H: 300 MHz; ^13^C: 75.5 MHz).

**Immobilisation of the first monomer:** Wang resin (485 mg, 0.315 mmol, capacity: 0.65 mmol/g) was swelled under argon with dry CH_2_Cl_2_ (1.5 mL) in an oven dried reaction vessel for 15 min. In a second dry vessel, ^13^C/^15^N-labelled Fmoc-Phe-OH (250 mg, 0.63 mmol) was dissolved in dry CH_2_Cl_2_ (3 mL) and MeIm (94 µL, 1.18 mmol). Afterwards MSNT (467 mg, 1.576 mmol) was added. The reaction mixture was shaken under argon until MSNT had completely dissolved. The amino acid solution was transferred via syringe to the resin and the suspension was flushed with argon. The sealed reaction vessel was gently agitated over night.

The beads were transferred to a syringe with a filter and washed with dry CH_2_Cl_2_ (5 ×). Some beads were dried in vacuo for the following colourimetric test with Dabcyl-COOH for the detection of polymer-supported OH groups [14]. (See Supporting Information File 1.)

**Coupling of further building blocks:** In case of quantitative conversion, the resin was washed with *N*,*N*-dimethylformamide (DMF, 5 ×) and treated three times (15 min, 10 min, 5 min) with a piperidine solution (25% in DMF). Then the resin was washed with *N*-methylpyrrolidone (NMP, 5 ×) and a solution of ^13^C/^15^N-labelled Fmoc-Leu-OH (250 mg, 0.69 mmol), HOBt*H_2_O (145 mg, 0.945 mmol) and DIC (145 µL, 0.945 mmol) in NMP (2.5 mL) was aspirated into the syringe for the next coupling step. After 3 h the resin was washed with NMP (5 ×) and a few beads were tested for quantitative conversion by the Kaiser test [16–17]. (See Supporting Information File 1.)

In case of a successful peptide extension, the Fmoc-protecting group was removed with piperidine (25% in DMF). Afterwards, the last coupling step was started by adding a solution of ^13^C/^15^N-labelled Fmoc-Met-OH (250 mg, 0.66 mmol), HOBt*H_2_O (145 mg, 0.945 mmol) and DIC (145 µL, 0.945 mmol) in NMP (2.5 mL). In case of quantitative coupling (Kaiser test), the Fmoc-protecting group was removed and the resin was washed with DMF (5 ×) and CH_2_Cl_2_ (5 ×). The beads were dried in the syringe under reduced pressure.

**Formylation of the *N*-terminus:** For the following formylation, ^13^C-labelled ethyl formate (2 mL) was aspirated and the syringe was shaken over night at room temperature. The quantitative conversion was proved by the Kaiser test, after washing the resin with CH_2_Cl_2_ (5 ×).

**Cleavage of the peptide from the solid support:** For a successful cleavage, it is highly recommended to dry the beads in vacuo for at least 3 h. The cleavage solution [TFA (1.88 mL), H_2_O (50 µL), EDT (50 µL) and TIS (20 µL)] was added to the syringe. After shaking (3 × 90 min), the liberated peptide was filtered off into Millipore™ water. This solution was lyophilised and the residue was purified by HPLC. Yield: 32.8 mg (23%).

HPLC conditions: Preparative: Reprosil AQ, 250 × 20, 10 µm, 0.1% TFA/MeCN (100:60), 10 mL/min; analytical: gradient: Reprosil AQ, 125 × 4.6 mm, 5 µm, 0.8 mL/min, t_R_ = 21.08 min; isocratic: Lichrospher RP8 (Merck), 125 × 4.0 mm, 5 µm, 0.1% TFA/MeCN (66:34), 0.8 mL/min, t_R_ = 5.07 min.

^13^C NMR (δ[ppm] 75.5 MHz, MeCN-d_3_): 173.5 (m, 1C), 172.6 (m, 1C), 171.8 (m, 1C), 162.8 (d, *J* = 12.8 Hz, 1C), 138.0 (m, 1C), 131.1-126.8 (m, 5C), 55.1-51.3 (m, 3C), 41.3 (t, *J* = 34.4 Hz, 1C), 37.8 (m, 1C), 32.4 (t, *J* = 35.5 Hz, 1C), 30.4 (m, 1C), 25.4 (m, 1C), 23.2 (m, 1C), 21.7 (m, 1C), 15.3 (s, 1C).

MS (ESI): *m*/*z* (%) = 460.3 (100.0) [M–H]^–^, ^13^C_21_H_31_^15^N_3_O_5_S calcd. 461.26.

## Supporting Information

Procedures for colourimetric resin tests (including synthesis and analytical data of Dabcyl-COOH), crystallisation protocol, ESI, ^13^C NMR and ^13^C/^15^N MAS-NMR spectra of f-MLF-OH (**1**) are available in the Supporting Information.

File 1 A practical synthesis of the ^13^C/^15^N-labelled tripeptide *N*-Formyl-Met-Leu-Phe, useful as a reference in solid-state NMR spectroscopy.

## References

[1] Rienstra C M, Hohwy M, Hong M, Griffin R G (2000). J Am Chem Soc.

[2] Rienstra C M, Hohwy M, Mueller L J, Jaroniec C P, Reif B, Griffin R G (2002). J Am Chem Soc.

[3] Rienstra C M, Tucker-Kellogg L, Jaroniec C P, Hohwy M, Reif B, McMahon M T, Tidor B, Lozano-Pérez T, Griffin R G (2002). Proc Natl Acad Sci U S A.

[4] Reif B, Hohwy M, Jaroniec C P, Rienstra C M, Griffin R G (2000). J Magn Reson.

[5] Jaroniec C P, Filip C, Griffin R G (2002). J Am Chem Soc.

[6] Jaroniec C P, Tounge B A, Herzfeld J, Griffin R G (2001). J Am Chem Soc.

[7] Wi S, Sinha N, Hong M (2004). J Am Chem Soc.

[8] McDermott A E (2004). Curr Opin Struct Biol.

[9] Lopez J J, Kaiser C, Shastri S, Glaubitz C (2008). J Biomol NMR.

[10] Tanaka F, Jones T, Kubitz D, Lerner R A (2007). Bioorg Med Chem Lett.

[11] Showell H J, Freer R J, Zigmond S H, Schiffmann E, Aswanikumar S, Corcoran B, Becker E L (1976). J Exp Med.

[12] Sheehan J C, Yang D-D H (1958). J Am Chem Soc.

[13] Blankemeyer-Menge B, Nimtz M, Frank R (1990). Tetrahedron Lett.

[14] Burkett B A, Brown R C D, Meloni M M (2001). Tetrahedron Lett.

[15] Chan W C, White P D (2004). Fmoc Solid Phase Peptide Synthesis: A Practical Approach.

[16] Kaiser E, Colescott R L, Bossinger C D, Cook P I (1970). Anal Biochem.

[17] Sarin V K, Kent S B H, Tam J P, Merrifield R B (1981). Anal Biochem.

[18] Jacobsen E J, Stelzer L S, Belonga K L, Carter D B, Im W B, Sethy V H, Tang A H, VonVoigtlander P F, Petke J D (1996). J Med Chem.

[19] Billault I, Courant F, Pasquereau L, Derrien S, Robins R J, Naulet N (2007). Anal Chim Acta.

[20] Jung S H, Ahn J H, Park S K, Choi J-K (2002). Bull Korean Chem Soc.

[21] Wang S-S (1973). J Am Chem Soc.

[22] Lu G-s, Mojsov S, Tam J P, Merrifield R B (1981). J Org Chem.

[23] Krebs A, Ludwig V, Pfizer J, Dürner G, Göbel M W (2004). Chem–Eur J.

[24] Suhartono M, Weidlich M, Stein T, Karas M, Dürner G, Göbel M W (2008). Eur J Org Chem.

[25] Carpino L A, Han G Y (1972). J Org Chem.

