# Supplementary material for: A practical synthesis of the 13C/15N-labelled tripeptide N-formyl-Met-Leu-Phe, useful as a reference in solid-state NMR spectroscopy
Source: Beilstein J Org Chem. 2008 Oct 13;4:35. doi: 10.3762/bjoc.4.35 (PMC2577282; doi:10.3762/bjoc.4.35)
Supplement: File 1 — A practical synthesis of the 13C/15N-labelled tripeptide N-Formyl-Met-Leu-Phe, useful as a reference in solid-state NMR spectroscopy. [file Beilstein_J_Org_Chem-04-35-s001.doc]

A practical synthesis of the ^13^C/^15^N-labelled tripeptide
*N*-formyl-Met-Leu-Phe, useful as a reference

in solid-state NMR spectroscopy

(Supporting Information)

Sven T. Breitung^1^, Jakob J. Lopez^2^, Gerd Dürner^1^, Clemens Glaubitz^2^,

Michael W. Göbel*^,1^ and Marcel Suhartono^1^

Address: ^1^Institute of Organic Chemistry and Chemical Biology, Goethe Universität Frankfurt, Max-von-Laue-Str. 7, D-60438 Frankfurt am Main, Germany

^2^Institute of Biophysical Chemistry, Goethe Universität Frankfurt, Max-von-Laue-Str. 9,
D-60438 Frankfurt am Main, Germany

**Mass spectrum of ^13^C/^15^N-labelled formyl-MLF-OH (1):**

MS (ESI): m/z (%) = 460.3 (100.0) [M–H]^–^, ^13^C_21_H_31_^15^N_3_O_5_S calcd. 461.26.


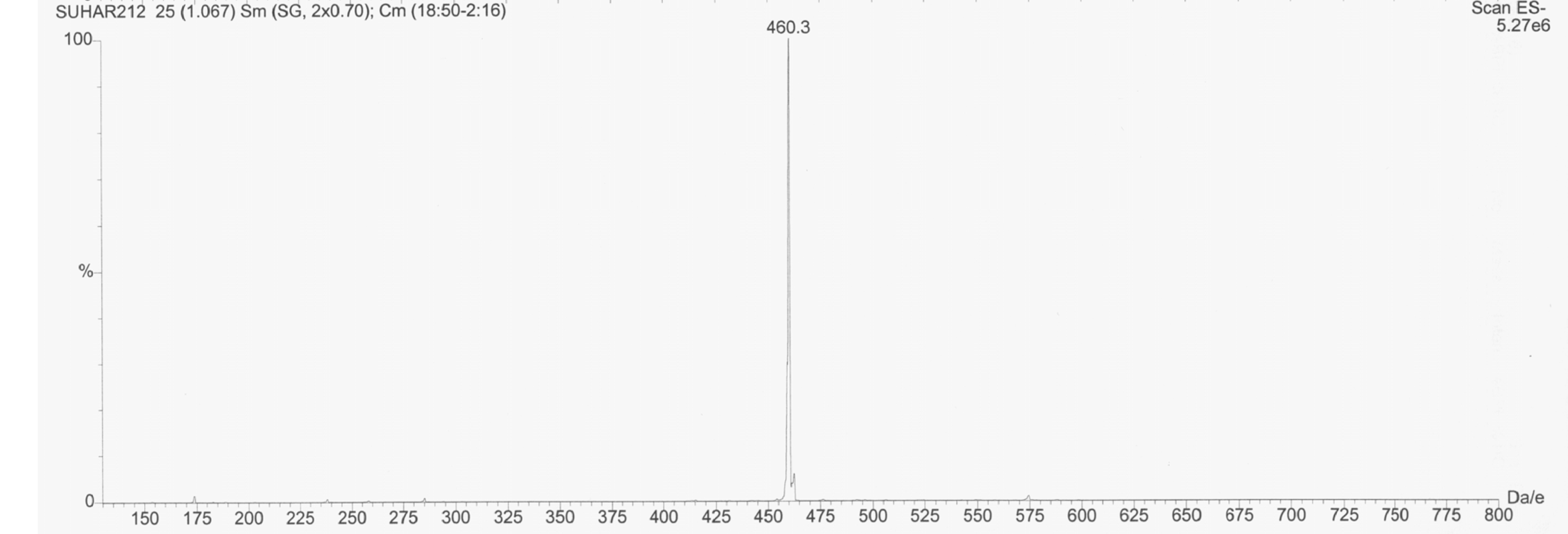


**^13^C NMR** (δ[ppm] 75.5 MHz, MeCN-d_3_): 173.5 (m, 1C), 172.6 (m, 1C), 171.8 (m, 1C), 162.8 (d, *J* = 12.8 Hz, 1C), 138.0 (m, 1C), 131.1–126.8 (m, 5C), 55.1–51.3 (m, 3C), 41.3 (t, *J* = 34.4 Hz, 1 C), 37.8 (m, 1 C), 32.4 (t, *J* = 35.5 Hz, 1C), 30.4 (m, 1C), 25.4 (m, 1C), 23.2 (m, 1C), 21.7 (m, 1C), 15.3 (s, 1C).

**Colourimetric test for the detection of polymer-supported alcohols:** A few dry resin beads (3 mg) were treated with NEt_3_ (10% in dry CH_2_Cl_2_, 300 µL) in an Eppendorf cap. Diphenyldichlorosilane (200 µL) was added. The cap was shaken for 10 min. The mixture was transferred to a syringe with a filter and the solution was discarded. The beads were washed twice with NEt_3_ (10% in dry CH_2_Cl_2_). A solution of Dabcyl-COOH (**7**) (4 mg) in DMF (500 µL) was drawn up and agitated for 10 min. If the resin was colourless after washing with DMF (5 ×) and DCM (until the solution is colourless), the coupling of the amino acid onto the polymer support was successful. Red coloured resin beads indicate free hydroxy groups.

**Kaiser test:** A few resin beads were treated with 3 drops of each of the following solutions [ninhydrine (5% in EtOH), phenol (80 g in 20 mL EtOH) and KCN (2 mL of a 0.001 M aqueous KCN solution in 98 mL pyridine] in an Eppendorf cap. The cap was heated (80 °C) in water for 5 min. If the resin was colourless, the coupling step was proved to be quantitative.

**Synthesis of 4-(4-dimethylamino-phenylazo)benzoic acid (Dabcyl) (7):**

|  | | | | |
| --- | --- | --- | --- | --- |
|  | | | | |
|  | **6** |  | **7** |  |
|  | | | | |

4-Aminobenzoic acid (**6**) (6.86 g, 50 mmol) was dissolved in a solution of HCl (6.25 mL, 12 M, 75 mmol) in water (15 mL) and the mixture was cooled with an ice bath. A solution of sodium nitrite (6.90 g, 100 mmol) in water (15 mL) and *N*,*N*-dimethylaniline (6.34 mL, 50 mmol) were added dropwise. The reaction mixture was stirred for 1 h at 0 °C. After warming up to room temperature, K_2_CO_3_ was added until pH 7 was observed. The resulting red precipitate was collected by filtration. The precipiate was washed thoroughly with water and then dried in vacuo. After recrystallisation from EtOH, Dabcyl (**7**) was obtained as a red solid (10.62 g, 78%).

R_f_ = 0.2 (EtOAc/*n*-hexane 2:1).

M.p. 275–278 °C; Lit: 271–273 °C [1].

^1^H NMR (δ[ppm] 250 MHz, DMSO-d_6_): 13.06 (bs, 1H, COO*H*), 8.07 (d, 2H, *J* = 8.75, 2×C^2^-*H*), 7.83 (d, 2H, *J* = 8.75, 2×C^3^-*H*), 7.82 (d, 2H, *J* = 9, 2×C^6^-*H*), 6.84 (d, 2H, *J* = 9, 2×C^7^-*H*), 3.07 (s, 6H, 2×C*H*_3_).

^13^C NMR (δ[ppm] 62.9 MHz, DMSO-d_6_): 166.7, 154.9, 152.9, 142.6, 130.8, 130.3, 125.1, 121.6, 111.5, 39.7.

IR (KBr): $\tilde{v}$= 3448 (w), 2366 (w), 1681 (s), 1594 (s), 1521 (m), 1420 (m), 1362 (s), 1298 (s), 1249 (m), 1134 (s), 943 (m), 862 (m), 820 (m), 774 (w), 726 (w), 692 (w).

Elemental analysis: C_15_H_15_N_3_O_2_ (269.30) calcd. C 66.90, H 5.61, N 15.60; found C 67.03, H 5.79, N 15.57.

**Crystallisation of ^13^C/^15^N-labelled formyl-MLF-OH (1):**

Samples for NMR were crystallised by first dissolving the lyophilised MLF in 2-propanol (typically, 5 mg in 2 mL), and letting the solution evaporate over night. Of the resulting powder, ca. 5 mg were transferred into a 3.2 mm rotor for NMR measurements.


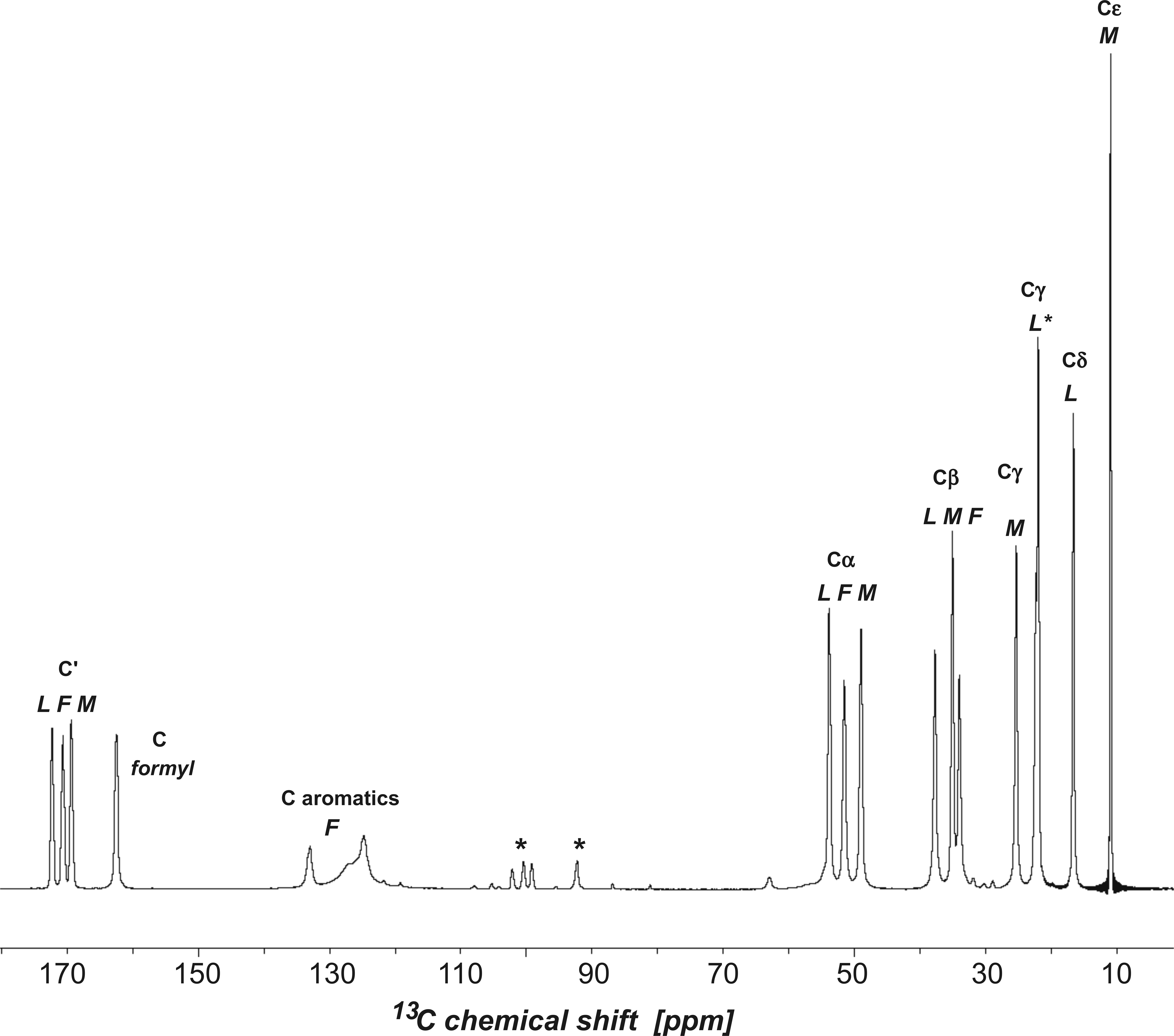
**^13^C/^15^N MAS-NMR on crystallised of ^13^C/^15^N-labelled formyl-MLF-OH (1):** All MAS NMR measurements were conducted on an 850 MHz Bruker Avance widebore spectrometer, with a rotor speed of 15 kHz, at a temperature of 250 K. The ^13^C NMR spectrum was acquired using a standard Hartman-Hahn ^1^H-^13^C cross polarisation experiment, with a contact pulse nutation frequency of 50 kHz, of 2 ms length. ^1^H 90° pulses were typically 3 μs long, and proton decoupling during acquisition was achieved with a SPINAL64 [2] block, operating at 83 kHz. ^15^N MAS spectra were acquired with the same parameters, but with a cross polarisation spinlock field of 35 kHz, and 3 ms contact time. For both, ^13^C and ^15^N spectra, 5000 scans were acquired.


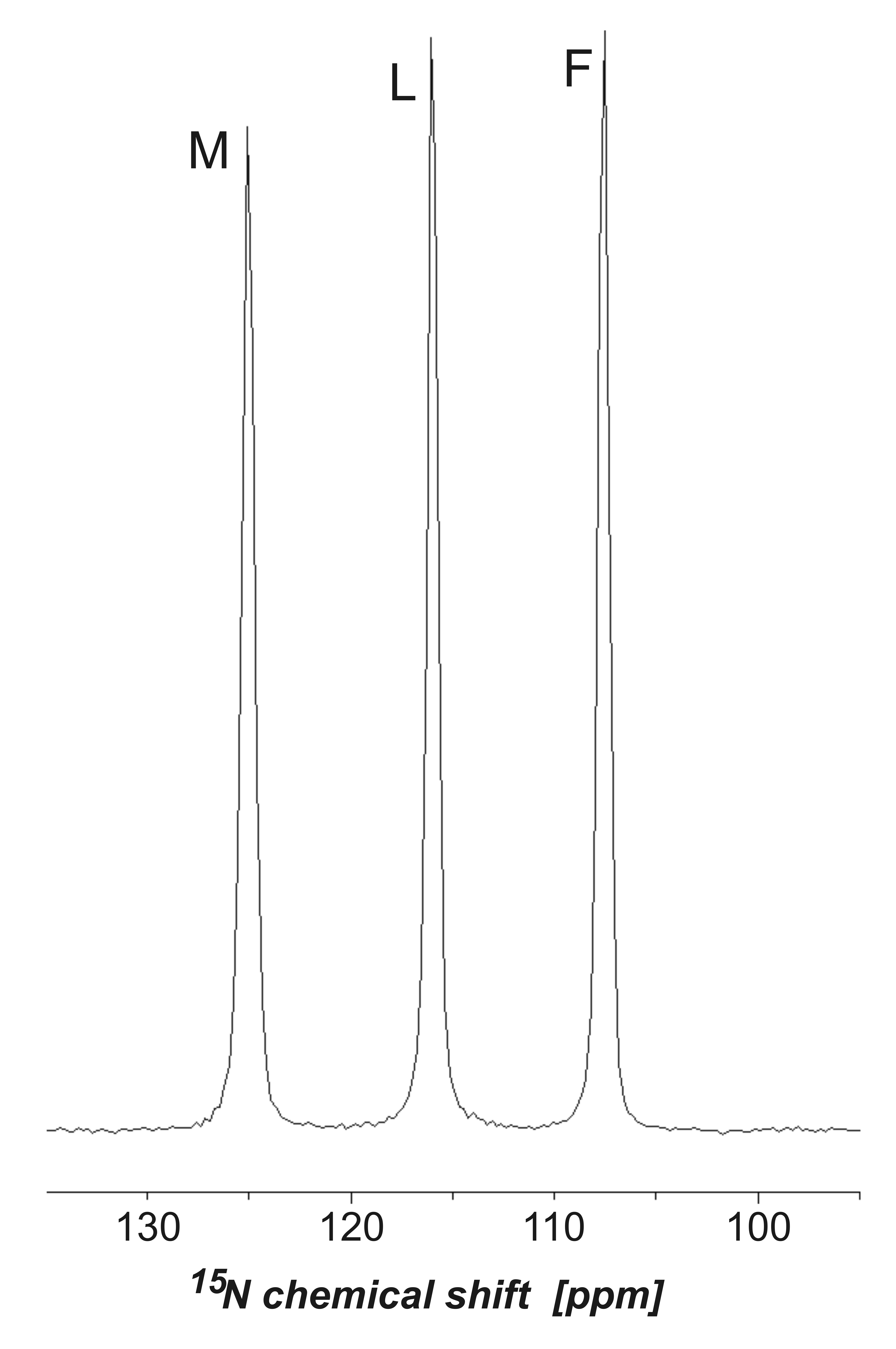


The resulting NMR spectra agree very well with published data, and indicate a high degree of microscopic order, as evidenced by a linebroadening which is smaller than 0.3 ppm in the ^13^C spectrum. Assignments were taken from Rienstra et al. [3].

**References**
